# A cancer vaccine based on fluorine-modified sialyl-Tn induces robust immune responses in a murine model

**DOI:** 10.18632/oncotarget.17646

**Published:** 2017-05-07

**Authors:** Chengcheng Song, Xiu-Jing Zheng, Chang-Cheng Liu, Yifa Zhou, Xin-Shan Ye

**Affiliations:** ^1^ State Key Laboratory of Natural and Biomimetic Drugs, School of Pharmaceutical Sciences, Peking University, Beijing 100191, China; ^2^ School of Life Sciences, Northeast Normal University, Changchun 130024, China

**Keywords:** cancer immunotherapy, glycoconjugate vaccine, tumor-associated carbohydrate antigen, fluoro-substituted STn, cross reaction

## Abstract

Development of an effective vaccine to target tumor associated carbohydrate antigens, aberrantly expressed on the cell surface of various carcinomas, is an appealing approach toward cancer immunotherapy. However, a major problem of carbohydrate antigens is their poor immunogenicity. Immunization with modified-carbohydrate antigens could improve the immunogenicity and induce cross reaction with the native carbohydrate antigens. In this study, we investigated the antitumor ability of three fluoro-substituted sialyl-Tn (STn) analogues (2, 3, 4) coupled to KLH (keyhole limpet hemocyanin) and studied the mechanism of tumor immunotherapy of the vaccines in a murine model of colon cancer. Vaccination with 4-KLH, in which the two *N*-acetyl groups of STn are substituted with *N*-fluoroacetyl groups, could remarkably prolong the survival of tumor-bearing mouse and resulted in a significant reduction in tumor burden of lungs compared with STn-KLH (1-KLH). The vaccine 4-KLH could provoke stronger cytotoxic T lymphocytes immune response, T helper (Th) cell-mediated immune response and an earlier-stage Th1 immune response than 1-KLH, thus breaking immune tolerance and generating a therapeutic response. The 4-KLH vaccine induced strong tumor-specific anti-STn antibodies which could mediate complement-dependent cytotoxicity and antibody-dependent cell-mediated cytotoxicity against human tumor cells. Moreover, in the absence of adjuvant, 4-KLH still elicited stronger immune responses than 1-KLH. Our data suggested that 4-KLH is superior in tumor prevention. The strategic hapten fluorination may be a potential approach applicable to the vaccines development for the cancer immunotherapy.

## INTRODUCTION

Tumor-associated carbohydrate antigens (TACAs), which are uniquely or excessively expressed on the surface of various tumors, are potential targets for the anti-cancer vaccine development [1–3]. TACAs usually possess poor immunogenicity and induce T cell-independent immune response. However, T cell-mediated immunity, which is related to immunoglobulin class switching from IgM to IgG, affinity maturation, and immunological memorization, is critical for cancer immunotherapy [4]. A widely-used strategy to address this problem is to conjugate TACAs to proper carriers such as proteins [4–6]. Although glycoconjugate anti-cancer vaccines have demonstrated promising therapeutic potentials at different stages of clinical trials [3, 7, 8], none of TACA-based vaccines has been approved by FDA yet. Some possible reasons are that these vaccines could not induce strong cytotoxic T lymphocytes (CTLs) immune response and could not sufficiently enhance the immunogenicity of TACAs [8]. Thus, new vaccine strategies that induce CTLs response and simultaneously enhance immunogenicity are desirable.

The expression of some TACAs in normal tissues, at a specific development stage, and their structural similarity to normal antigens make them more likely to be recognized as ‘self’ by immune system, leading to immune tolerance [2]. TACAs have been chemically modified to make them more ‘foreign’ to break the tolerance [1, 9]. The chemical modification-based antitumor vaccines involve two strategies: the metabolic oligosaccharide engineering (MOE)-based immunotherapy which contains administration of biosynthetic precursors of modified TACAs and then immunization with the modified carbohydrate antigens [10–12], and the cross-reactivity-based immunotherapy which only needs immunization with the modified carbohydrate antigens. The latter is simpler in procedure and may induce fewer side effects, compared with the MOE strategy. Some chemical modifications of the TACAs based on cross-reactivity strategy can break the immune-tolerance and enhance the immunogenicity of the TACAs, for instance, GD2- and GD3-lactone in patients with melanoma, and *N*-propylated polysialic acid in patients with small cell lung cancer, got promising results in clinical trials [13–15]. In preclinical studies, some modified-TACAs induced strong cross-reactive immune responses in mice [1, 16–22]. Chemical modifications of the carbohydrate antigens based on cross-reactivity strategy were also applied to other diseases such as HIV [23] and meningitis [24]. Meanwhile, the extent of modification must be precisely controlled to make the antigen immunogenic enough to break tolerance and be capable of inducing cross-reactive antibodies at the same time [4].

Fluoro-substituted compounds have been applied in medicinal chemistry owing to the unique properties of fluorine, such as its absence in most organisms, its comparable size to hydrogen, its high bond strength to carbon, its inductive effect and the ‘polar hydrophobicity’ [25, 26]. Incorporation of fluorine atom is frequently used to generate high-affinity ligand binding [27] and has been also applied in the field of vaccine for enhancing immune response [4]. Indeed, immune response in part is based on the T-cell receptor (TCR) recognition of antigenic molecules bound and presented by the major histocompatibility complex (MHC) [28]. Enhancing TCR affinity via peptide modifications has been discussed as a means to help break immunological tolerance and improve the antigenicity of antigens [29, 30]. It has been proved that fluorinated antigen is one of effective approaches to enhance TCR affinity without significantly perturbing its composition or structure [31]. Thus, some fluoro-modified antigens were prepared to meet the structural and functional requirements of vaccines, and among these investigations, some analogues demonstrated significant improvement of the immunogenicity as vaccines. For example, fluorine-containing cocaine haptens possess potent cocaine affinity, and can elicit higher concentration of antibodies than the original structure succinyl norcocaine [28]. In our previous studies, some fluorinated modifications of TACAs, including Thomsen-nouveau (Tn), sialyl Tn (STn), TF and ganglioside GM3, were conjugated to proteins, improving the immunogenicity of the antigens and inducing the strong cross-reactive immune response in mice [17–20].

The aim of the present work is to evaluate the efficacy of the fluorine-modified vaccines in the treatment of tumors and analyze the structure-activity relationships for the discovery of more effective vaccines. To achieve this, STn, a sialylated disaccharide, was chosen as the target antigen. The STn was abundantly expressed on various tumors such as pancreas, breast, prostate ovarian, and colorectal cancers. Theratope was designed by coupling a synthetic native STn to the protein carrier keyhole limpet hemocyanin (KLH). It failed in phase III clinical trial, only modest clinical efficacy was achieved when patients were treated in conjugation with hormone therapy. Some possible reasons for the failure of Theratope could be insufficient enhancement of STn immunogenicity and did not induce strong T-cell-mediated immune response [8]. In the previous study [17], we have demonstrated that the fluorine-containing STn derivatives (compounds 2, 3, and 4) are much more immunogenic than the native STn in healthy mice. We are interested in anti-tumor effects of the fluorine-containing STn conjugates in tumor-bearing mice. In this study, a tumor-bearing model was constructed with a murine colon cancer cell line CT-26 for investigating the antitumor activity of the fluorine-modified vaccines and the mechanism of tumor immunotherapy of the vaccines in the presence or absence of adjuvant. Our results showed that immunization with 4-KLH in combination with adjuvant can elicit potent CTL immune response and T helper (Th) cell-mediated immune response, and is effective in reversing tolerance and generating a therapeutic response. The vaccine 4-KLH could induce strong anti-STn IgG antibodies capable of specifically recognizing STn-expressing tumor cells. Moreover, these antibodies were able to mediate complement-dependent cytotoxicity (CDC) and antibody-dependent cell-mediated cytotoxicity (ADCC) against STn-positive human tumor cells. In the absence of adjuvant, the vaccine 4-KLH still evoked stronger immune responses than STn-KLH (1-KLH). It has been found that the fluoro-substitution in both *N*-acetyl groups of the STn is critical for inducing optimal immune responses, providing some important considerations for further investigation and development of carbohydrate-based anticancer vaccines.

## RESULTS

### Antigen design and tumor challenge studies

**Figure 1:**
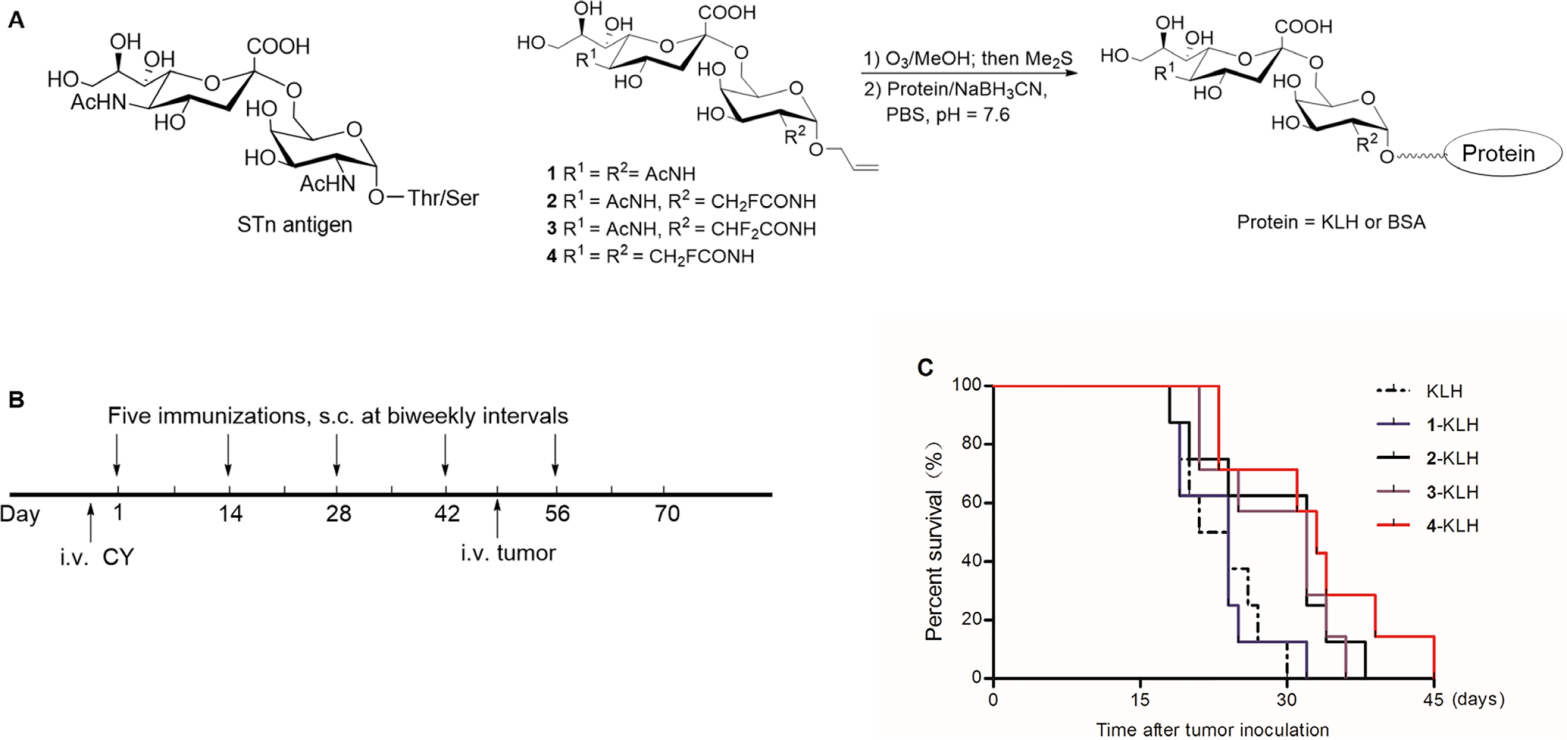
Fluoro-substituted STn vaccines induce efficient antitumor immunotherapy in the presence of adjuvant (**A**) Preparation of the glycoconjugates. (**B**) Schematic representation of the survival study design and immune scheme. One day before the immunization schedule, mice were given intravenous (i.v.) injection of cyclophosphamide (CY) at a dose of 100 mg/kg per mouse. The glycoconjugates in the presence of adjuvant were immunized five times at biweekly intervals and animals were challenged via the tail vein 7 days after the 4th immunization with 2 × 10^5^ CT-26 cells. (**C**) Survival time of tumor-bearing mice. KLH group (*n* = 8), 1-KLH group (*n* = 8), 2-KLH group (*n* = 8), 3-KLH group (*n* = 7) and 4-KLH group (*n* = 7). There is significant difference between 4-KLH group and 1-KLH group. The abscissa represents the time after tumor inoculation.

To overcome the low immunogenicity of STn and elicit a powerful immunological response, we investigated the effect of some fluorine-modified STn compounds. STn (Figure 1A, compound 1) and the modified derivatives 2, 3, 4 were synthesized [17]. Glycoconjugate vaccines 1-KLH, 2-KLH, 3-KLH and 4-KLH were prepared by reductive amination reaction (Figure 1A).

The detail of the immunization program was shown in Figure 1B. Immunization with 4-KLH significantly extended the survival of tumor-bearing mouse compared with 1-KLH (*p* = 0.02) or treatment with KLH (*p* = 0.006) which did not contain the STn epitope (Figure 1C). Vaccination with 2-KLH and 3-KLH could prolong survival time compared to the KLH group and 1-KLH group, without significant difference between them. No significant difference was seen in the survival of mice vaccinated with 1-KLH compared to the KLH group.

**Figure 2:**
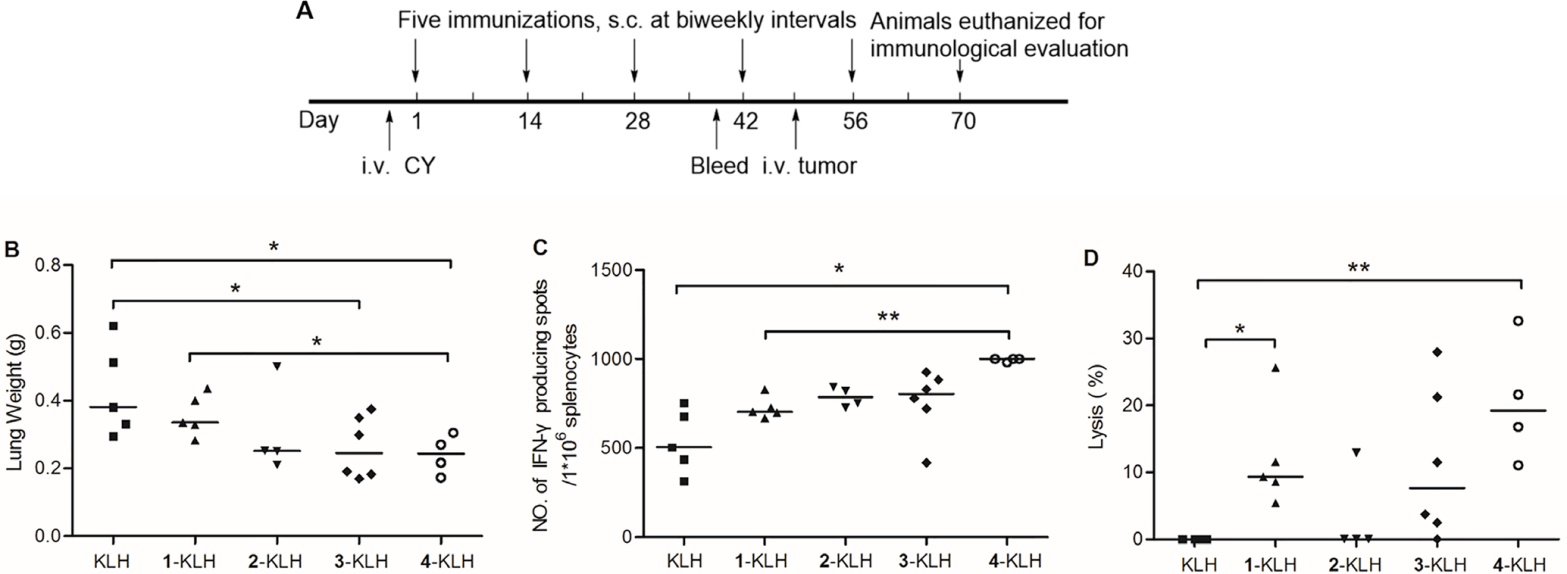
Fluoro-substituted STn vaccines improve the cellular immune response in the presence of adjuvant (**A**) Schematic representation of the immune scheme. KLH, 1-KLH, 2-KLH, 3-KLH and 4-KLH in the presence of adjuvant were immunized five times at biweekly intervals and animals were challenged via the tail vein 7 days after the 4th immunization with 2 × 10^5^ CT-26 cells. Three weeks after tumor challenge, animals were euthanized, lungs and splenocytes were separated from each mouse for immunological evaluation. (**B**) Lungs were weighted and assessed for tumor load. Results are expressed as the median of individual. (**C**) ELISPOT IFN-γ-releasing splenocytes assay. (**D**) CTL assay: *in vitro* cytotoxicity of splenocytes obtained from each group against CT-26 cells. Results are presented as median values for groups of 4–6 mice. *p < 0.05 and **p < 0.01.

Two weeks after the last immunization, the mice were euthanized for immunological evaluation. The detailed experimental scheme was shown in Figure 2A. Mouse tumor burden in lungs was used as reference to evaluate the effect of vaccine on tumor burden produced by i.v. injection of CT-26 cells to BALB/c mice. Tumor burden in lungs was measured as whole organ weight, significantly decreased in the 3-KLH and 4-KLH vaccinated mice, as compared with the KLH group (Figure 2B). The 2-KLH injected animals showed reduction in tumor burden of lungs compared to treatment with KLH, but there was no statistically significant difference between them. Immunization with 4-KLH resulted in a remarkable reduction in tumor burden of lungs compared to treatment with 1-KLH.

### Evaluation of the mouse T cell response to vaccination

To investigate the cellular immunity responses the vaccines induced, correlation analyses including the number of IFN-γ-producing splenocytes cells and the ability of these cells to lyse cells [32] were performed.

Antigen-specific IFN-γ-producing T cells were examined by an ELISPOT assay. As revealed in Figure 2C, there was a significant increase in the quantity of IFN-γ-releasing splenocytes after 4-KLH immunization compared with the mice vaccinated with 1-KLH or KLH. The IFN-γ-producing frequency of splenocytes in the mice, which were treated with 2-KLH and 3-KLH, slightly increased without significant difference. Immunization with 4-KLH might establish the strong T cell-mediated immunity, which is critical to effective cancer immunotherapy.

To assess the ability of the vaccine candidates to activate CTLs, splenocytes cells from immunized mice were isolated and incubated with CT-26 cancer cells. As shown in Figure 2D, the isolated splenocytes from 1-KLH and 4-KLH immunized mice demonstrated significantly higher cytotoxicity to CT-26 cell, compared with that of the KLH group. CTLs activated by the vaccine 4-KLH exhibited greater cytotoxicity compared with 1-KLH, further demonstrating 4-KLH could evoke stronger T cell-mediated immunity than 1-KLH. The mice immunized with 2-KLH and 3-KLH exhibited a reduced lytic activity.

### Evaluation of the antibody response to vaccination

**Figure 3:**
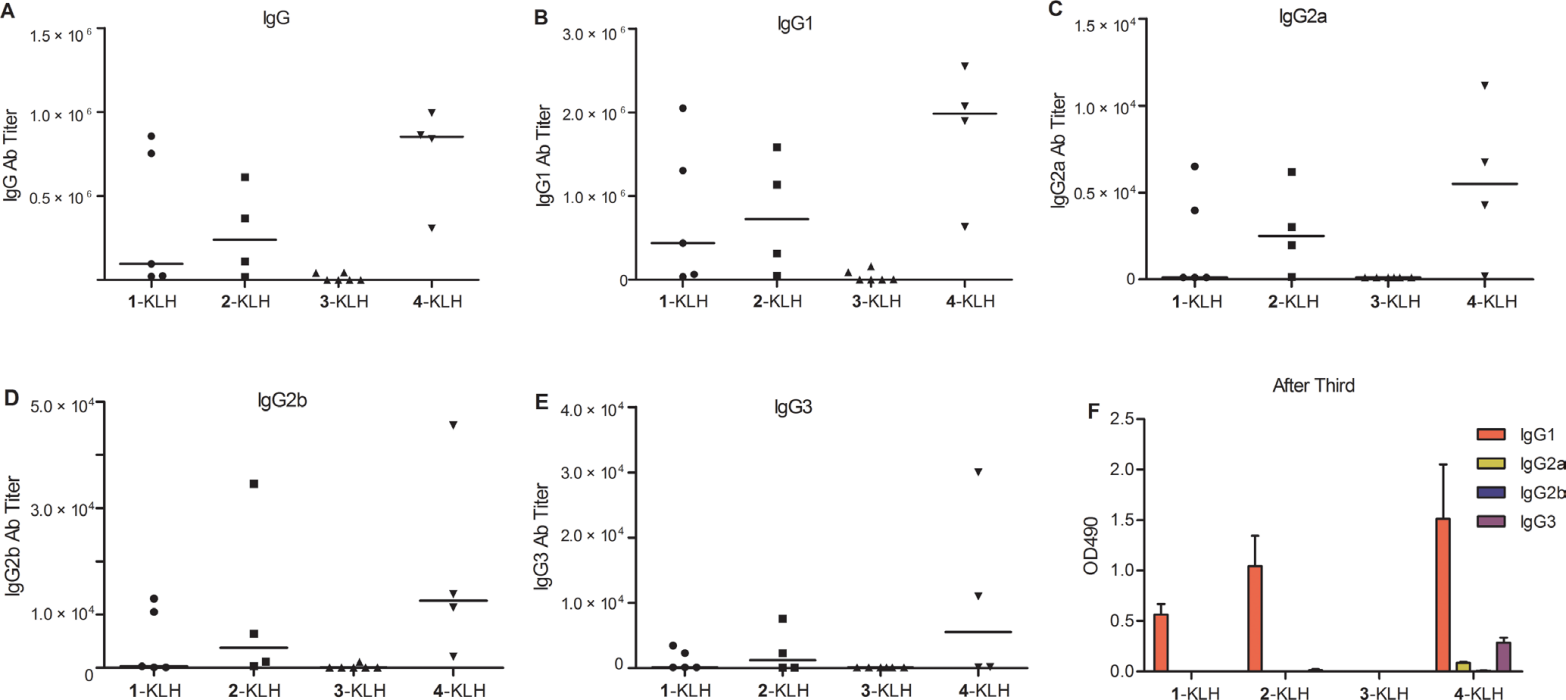
Fluoro-substituted STn vaccines improve the antibody response in the presence of adjuvant 1-KLH, 2-KLH, 3-KLH and 4-KLH in the presence of adjuvant were immunized five times at biweekly intervals and animals were challenged via the tail vein 7 days after the 4th immunization with 2 × 10^5^ CT-26 cells. The mice were bled by tail vein on day 13 after the third immunization on day 14 after the fifth immunization. (**A**–**E**) ELISA tests of anti-STn antibody titers after 5th immunization with antigen. Each data point represents the titer of an individual mouse and the horizontal lines indicate the median for the group of mice. (**F**) IgG subtypes after immunized with 1-KLH, 2-KLH, 3-KLH and 4-KLH by ELISA with a 1:1000 dilution of pooled sera. The pooled sera obtained from 13 days after the 3rd vaccination. OD490 = optical density at 490 nm. The results in (A–E) are representative of three independent experiments. The results in (F) represent the mean ± standard error of measurement (SEM) of at least two experiments.

Anti-STn or anti-modified-STn antibody titers were detected by coating ELISA (enzyme-linked immunosorbent assay) plates with 1-BSA or the modified-STn-BSA, using the pooled antisera of all immunized mice after the third or the fifth immunization (Table 1). We found that 2-KLH and 4-KLH provoked a strong STn-specific immune response and elicited higher titers of anti-STn IgG antibodies than 1-KLH. The KLH control group showed no significant titer to STn. The anti-STn IgG titer for individual mouse was detected. The IgG level for 4-KLH was higher than that for 1-KLH (Figure 3A and Supplementary Figure 1). Meanwhile, the modified-STn conjugates 2-KLH and 4-KLH produced high anti-modified-STn antibody titers with reasonable cross-recognition efficiency, resulting in the increase of anti-STn IgG antibodies. By contrast, immunization with 3-KLH elicited less anti-STn and anti-modified-STn IgG antibody titers than that of 1-KLH, reflecting the weak immunogenicity of 3-KLH and the poor cross-recognition efficiency. In modified-STn-KLH groups, there were no detectable IgM antibodies against STn in the sera at a dilution of 1:100. In 1-KLH group, after the fifth immunization, slight anti-STn IgM antibodies were detected (Table 1). For tumor immunotherapy, IgG responses are more desirable than IgM on account of their properties such as affinity maturation and immunological memory [2, 33].

**Table 1:** Immunological results after vaccination with synthetic carbohydrate conjugates in the presence of adjuvant

Vaccine	ELISA titer anti-STn				ELISA titer anti-modified-STn
	After third		After fifth		After fifth
	IgG	IgM	IgG	IgM	IgG
KLH	< 100	< 100	< 100	< 100	------
1-KLH	15210	< 100	347080	126	347080
2-KLH	128672	< 100	444915	< 100	770080
3-KLH	< 100	< 100	14479	< 100	34026
4-KLH	532951	< 100	1047799	< 100	1739261

KLH, 1-KLH, 2-KLH, 3-KLH and 4-KLH in the presence of adjuvant were immunized five times at biweekly intervals and animals were challenged via the tail vein 7 days after the 4th immunization with 2 × 10^5^ CT-26 cells. ELISA anti-STn or anti-modified-STn antibody titers in the presence of adjuvant were determined by coating ELISA plates with the 1-BSA or modified-STn-BSA. Using the pooled antisera of all immunized mice obtained from 13 days after the third immunization or 14 days after the fifth immunization, and the antibody titer was defined as the highest dilution showing an absorbance of 0.1, after subtracting background. Anti-STn IgM titers could not be detected when diluted 1:100 (expressed as < 100). The results are representative of three independent experiments.

Glycoconjugates 2-KLH and 4-KLH evoked robust IgG antibody responses, and subtypes of the IgG indicated a mixed Th1/Th2 response (Figure 3B–3E), because the generation of IgG1 antibodies is usually associated with Th2 responses, whereas activated Th1 cells might generate a predominant IgG2a response [34, 35]. The median of individual anti-STn IgG subtype antibody titers of 2-KLH and 4-KLH were higher than that of 1-KLH, suggesting that a broad and balanced IgG immune response has been elicited, and the pooled antisera of all immunized mice revealed that 4-KLH produced Th1 response much earlier than 1-KLH (Figure 3F, Supplementary Figure 2). Immunization with the modified-STn carbohydrate conjugate 4-KLH could cause stronger IgG3 antibody response than that of 1-KLH (Figure 3E), which is a typical anti-carbohydrate response [36].

### Recognition and killing of human tumor cells by antibodies from vaccinated mice

**Figure 4:**
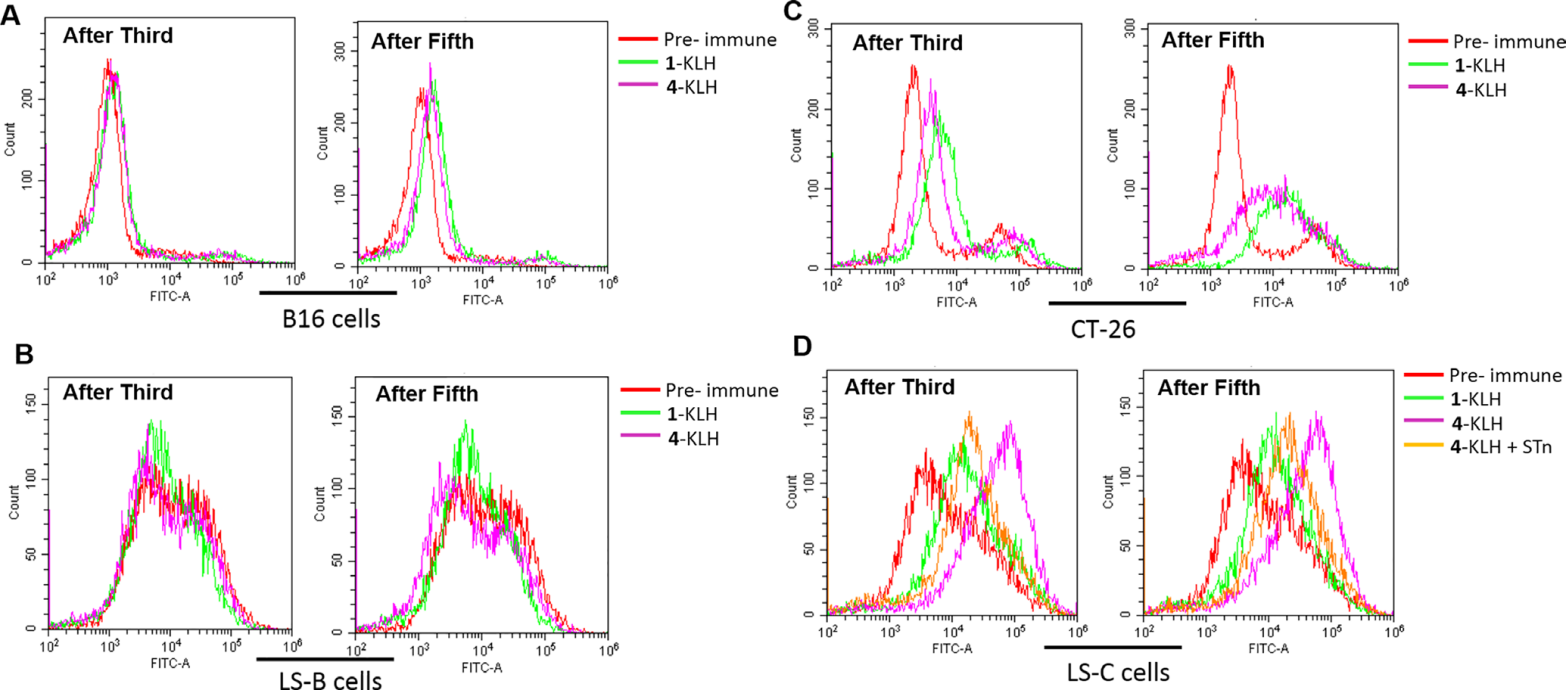
The antisera elicited by 4-KLH vaccine in the presence of adjuvant can recognize cancer cells expressing the native STn antigen 1-KLH and 4-KLH were immunized five times at biweekly intervals and animals were challenged via the tail vein 7 days after the 4th immunization with 2 × 10^5^ CT-26 cells. The mice were bled by tail vein on day 13 after the third immunization and on day 14 after the fifth immunization. Serological IgG analysis results on B16 cells (**A**), LS-B cells (**B**), CT-26 cells (**C**) and LS-C cells (**D**) after the third and the fifth immunization with 1-KLH and 4–KLH by flow cytometry. (D) The competitive inhibition of serum recognition of LS-C cells using carbohydrate STn (compound 1) as inhibitor. The results are representative of at least two independent experiments.

To evaluate the antitumor immunotherapy potential of antibodies elicited in mice, flow cytometry was used to analyze their ability to specifically recognize the tumor cells. Pre-immune sera from mice and STn negative tumor cells were used as negative control, almost no background reactivity was found (Figure 4A–4B), and the antisera elicited by 4-KLH and 1-KLH reacted strongly with the CT-26 and LS-C cells, after both 3rd and 5th vaccination (Figure 4C–4D). The post-immunization sera from 4-KLH vaccinated mice showed an increase of reactivity with LS-C cells, compared with the antisera elicited against 1-KLH. To confirm that the antisera elicited by 4-KLH recognized LS-C tumor cells was specific for STn, serum was first mixed with STn (compound 1) before being added to the cells. The results showed that the addition of STn significantly inhibited the serum recognition of tumor cells (Figure 4D). These results demonstrated that the antibodies elicited by 4-KLH can specifically recognize the tumor cells expressing the native STn antigen, which is vital for potent cancer immunotherapy.

**Figure 5:**
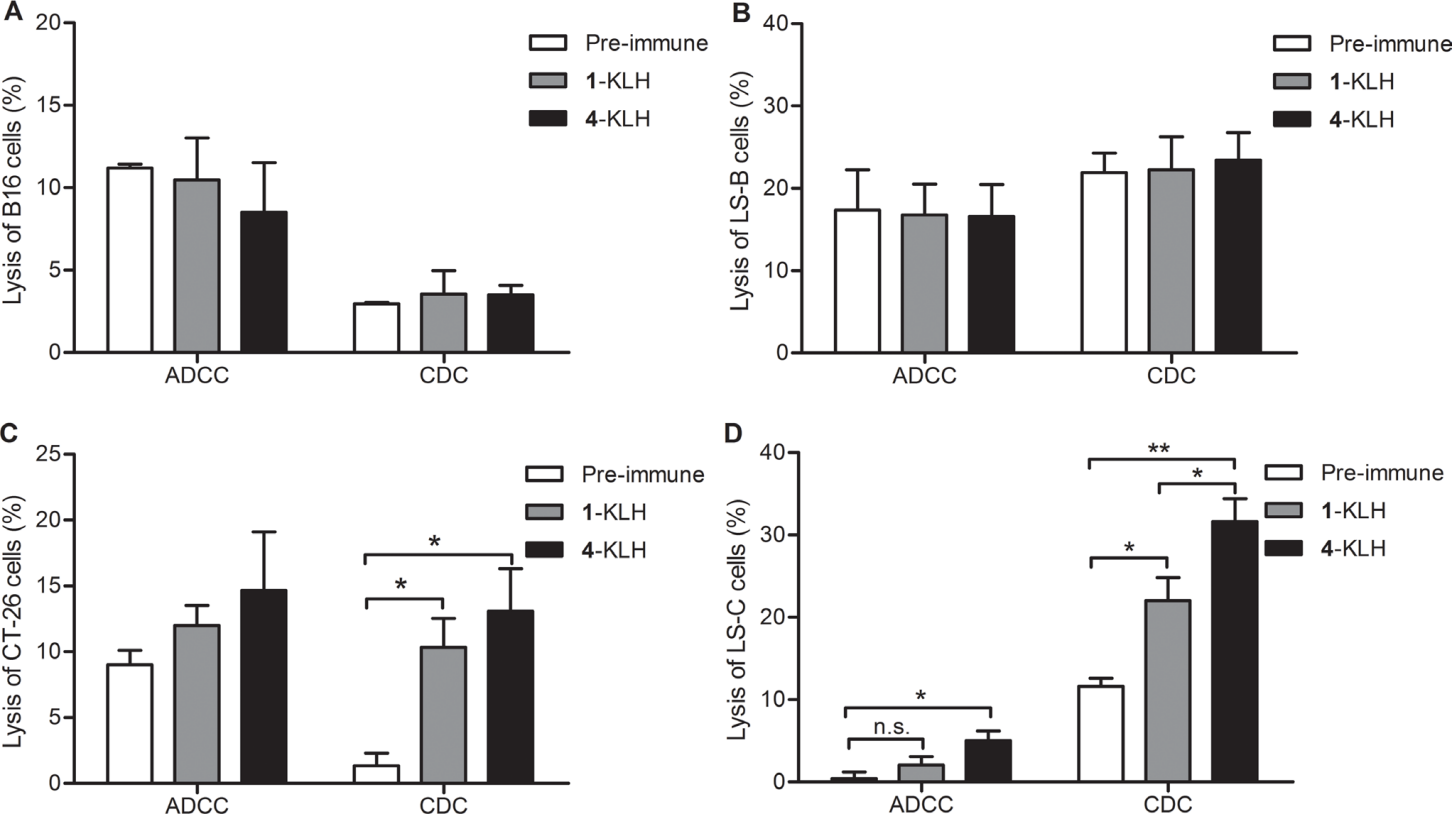
The antisera elicited by 4-KLH vaccine in the presence of adjuvant can kill cancer cells expressing the native STn antigen by ADCC and CDC assays Target cell B16 cells (**A**), LS-B cells (**B**), CT-26 cells (**C**) and LS-C cells (**D**) were incubated with the pooled sera from different vaccinated mice after 5th immunizations for 2 h at 37°C, and then incubated with effectors. Cell lysis was evaluated by LDH assay. The results represent the mean ± SEM of at least three experiments. *p < 0.05, **p < 0.01 and n.s. = no significance.

To evaluate whether 4-KLH and 1-KLH-provoked anti-STn antibodies are functional *in vitro*, we assessed their capacity to activate the pathway of ADCC and CDC. The STn-negative cancer cells and the antisera obtained from the pre-immune were used as negative control. For STn-negative tumor cells, there was no difference in tumor cell lysis rate between the 1-KLH group and the 4-KLH group as compared with the pre-immune serum (Figure 5A–5B). The antisera generated by immunization with 4-KLH significantly increased cancer cell lysis compared with the pre-immune serum, and antibodies elicited by 1-KLH were less efficacious in cell lysis compared with 4-KLH for STn-expressing cancer cells (Figure 5C–5D), highlighting the importance of antigen modification for relevant antigenic responses.

### Evaluation of the immune responses of the vaccine in the absence of adjuvant

In order to exclude that a strong immune response to the vaccines was induced by adjuvant, the mice were immunized with glycoconjugates in the absence of adjuvant. The effectiveness of anti-tumor was evaluated by determining the weight of lungs. The T cell-mediated immune responses were evaluated by determining the number of IFN-γ-producing splenocytes cells. The humoral immune responses were evaluated by titers of STn-specific antibodies and the ability of the antisera to lyse STn-bearing tumor cells.

**Figure 6:**
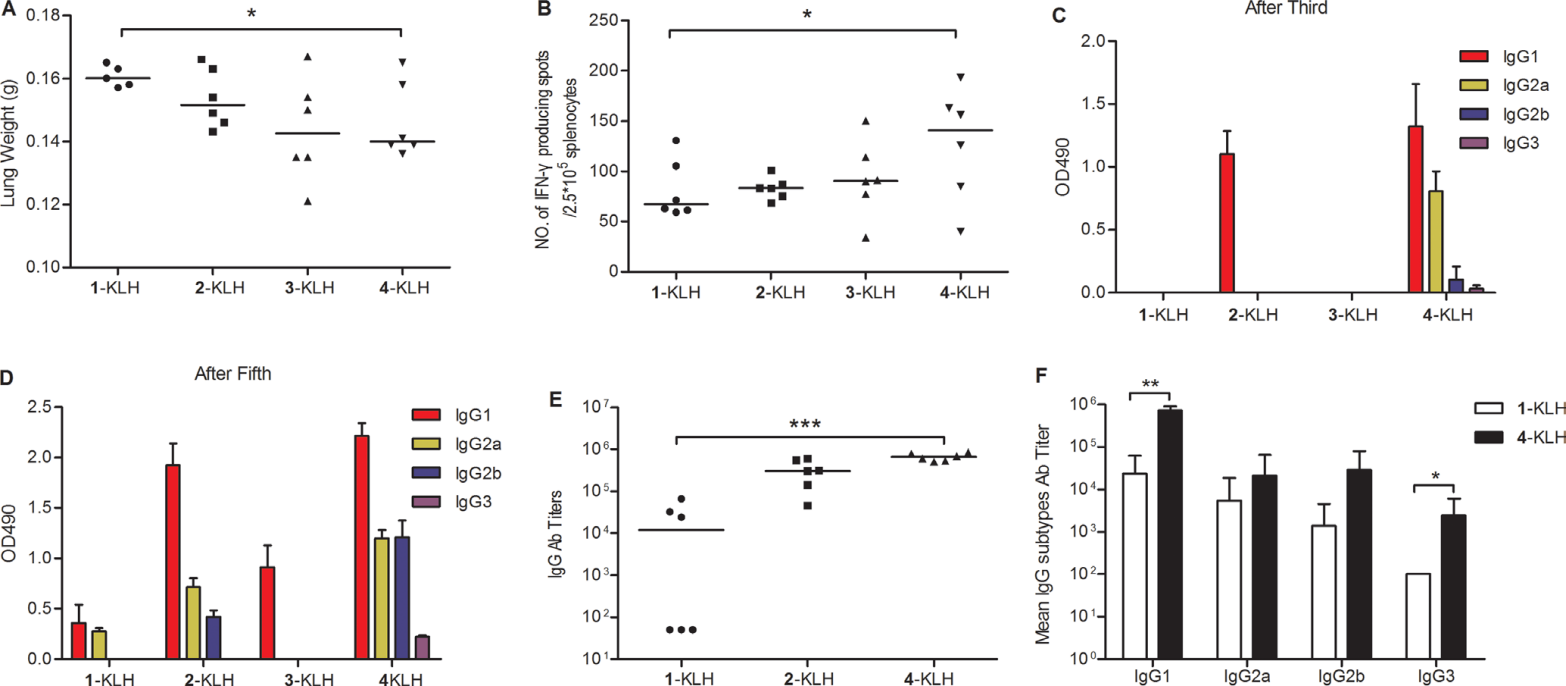
Fluoro-substituted STn vaccines induce efficient antitumor immunotherapy, T cell-mediated immunity and antibody immune response in the absence of adjuvant (**A**) 1-KLH, 2-KLH, 3-KLH and 4-KLH in the absence of adjuvant were immunized five times at biweekly intervals and animals were challenged via the tail vein 7 days after the 4th immunization with 1.5 × 10^5^ CT-26 cells. Two weeks after tumor challenge, animals were euthanized, lungs were weighted and assessed for tumor load. Results are presented as median values for groups. (**B**) ELISPOT IFN-γ-releasing lymphocyte assay. ELISPOT assays of the number of IFN-γ-releasing splenocytes among 2.5 × 10^5^ splenocytes derived from each mouse. Results are presented as median values for groups of 6 mice. (**C**–**D**) IgG subtypes after immunized with 1-KLH, 2-KLH, 3-KLH and 4-KLH without adjuvant by ELISA with a 1:1000 dilution of pooled sera. (**E**–**F**) ELISA anti-STn antibody titers after 5th immunization. Results are presented as median values for groups of 6 mice (E) or as the mean of individual ± standard deviation (SD) (F). The results in (A-B, E-F) are representative of three independent experiments. *p < 0.05, **p < 0.01 and ***p < 0.001. The results in (C–D) represent the mean ± SEM of at least two experiments.

Immunization with 4-KLH led to a significant reduction in tumor burden of lungs compared to treatment with 1-KLH (Figure 6A). The number of IFN-γ-releasing splenocytes after 4-KLH immunization was significantly higher than that of mice vaccinated with 1-KLH (Figure 6B). In the absence of adjuvant, immunization with 4-KLH was still able to inhibit the growth of tumor and establish strong T cell-mediated immunity.

**Figure 7:**
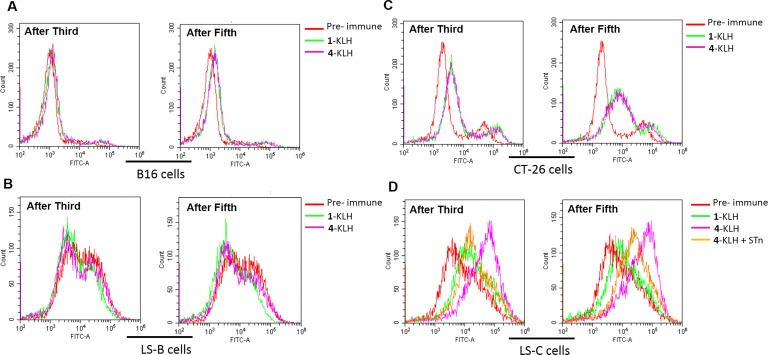
The antisera elicited by 4-KLH vaccine in the absence of adjuvant can recognize cancer cells expressing the native STn antigen 1-KLH and 4-KLH in the absence of adjuvant were immunized five times at biweekly intervals and animals were challenged via the tail vein 7 days after the 4th immunization with 1.5 × 10^5^ CT-26 cells. The mice were bled by tail vein on day 13 after the third immunization and on day 7 after the fifth immunization. Serological IgG analysis results on B16 cells (**A**), LS-B cells (**B**), CT-26 cells (**C**) and LS-C cells (**D**) after the third and fifth immunization with 1-KLH and 4-KLH by flow cytometry. (D) The competitive inhibition of serum recognition of LS-C cells using carbohydrate STn (compound 1) as inhibitor. The results are representative of two independent experiments.

**Figure 8:**
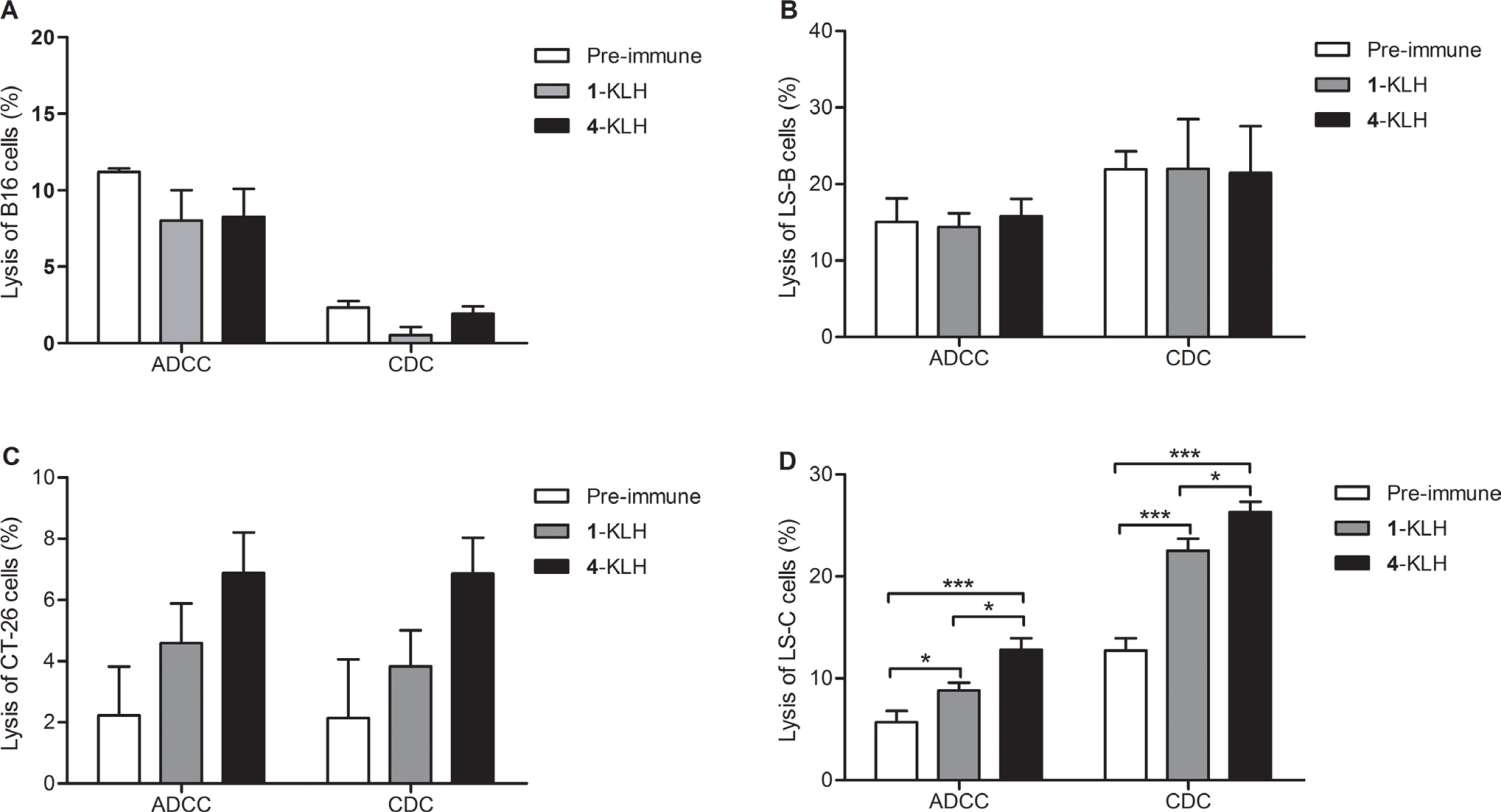
The antisera elicited by 4-KLH vaccine in the absence of adjuvant can kill cancer cells expressing the native STn antigen by ADCC and CDC assays Target cell B16 cells (**A**), LS-B cells (**B**), CT-26 cells (**C**) and LS-C cells (**D**) were incubated with the pooled sera from different vaccinated mice after 5th immunizations for 2 h at 37°C, and then incubated with effectors. Cell lysis was evaluated by LDH assay. The results represent the mean ± SEM of at least three experiments. *p < 0.05 and ***p < 0.001.

Immunization with 2-KLH and 4-KLH induced a strong antibody immune response, elicited higher anti-STn and anti-modified-STn IgG antibodies than that of 1-KLH (Table 2). ELISA tests with pooled antisera revealed that 4-KLH produced Th1 response much earlier than 1-KLH (Figure 6C–6D), and the subtypes of the IgG indicated a mixed Th1/Th2 response. The anti-STn IgG level for individual mouse serum from 4-KLH obviously increased compared to that from 1-KLH. The anti-STn IgG level for individual mouse serum from 2-KLH was higher than 1-KLH without significant difference (Figure 6E). So we chose the group of 4-KLH for further analysis of IgG antibody subtypes. The anti-STn IgG1, IgG3, IgG2a and IgG2b antibody titers of 4-KLH were significantly higher than that of 1-KLH (Figure 6F). The flow cytometry assay showed that the antisera elicited by 4-KLH did not react with the negative tumor cells (Figure 7A–7B) and could specifically and strongly react with the LS-C and CT-26, both after the third and after the fifth vaccination (Figure 7C–7D). For ADCC and CDC assays, the pooled sera from 4-KLH and 1-KLH showed no difference in the lysis rate of STn-negative tumor cells compared with pre-immune serum (Figure 8A–8B). The 4-KLH serum showed a significant increase in the lysis rate of STn-expressing tumor cells compared with the pre-immune serum and the 1-KLH serum (Figure 8C–8D).

**Table 2:** Immunological results after vaccination with synthetic carbohydrate conjugates in the absence of adjuvant

	ELISA titer anti-STn		ELISA titer anti-modified-STn
	IgG	IgM	IgG
KLH	< 100	< 100	------
1-KLH	15309	< 100	15309
2-KLH	293812	< 100	588662
3-KLH	7384	< 100	14794
4-KLH	555186	< 100	1061530

KLH, 1-KLH, 2-KLH, 3-KLH and 4-KLH in the absence of adjuvant were immunized five times at biweekly intervals and animals were challenged via the tail vein 7 days after the 4th immunization with 1.5 × 10^5^ CT-26 cells. ELISA anti-STn or anti-modified-STn antibody titers after 5th immunization in the absence of adjuvant were determined by coating ELISA plates with the 1-BSA or modified-STn-BSA. Using the pooled antisera of all immunized mice obtained from 7 days after the fifth immunization, and the antibody titer was defined as the highest dilution showing an absorbance of 0.1, after subtracting background. Anti-STn IgM titers could not be detected when diluted 1:100 (expressed as < 100). The results are representative of three independent experiments.

## DISCUSSION

Enhancing the immunogenicity of TACAs is one of the effective ways to improve cancer immunotherapy. Previously, we found that some fluorinated STn antigens are significantly more immunogenic than their natural counterparts [17]. Herein, we analyzed three modified-STn and STn immunogens, 2-KLH, 3-KLH, 4-KLH and 1-KLH, in an experimental lung metastases murine model, and revealed that immunization with three modified-STn-KLH immunogens could induce anti-tumor protection, especially 4-KLH, which led to a significant increase in the survival time of tumor-bearing mouse and a significant reduction in tumor burden of lungs compared with 1-KLH. Meanwhile, we reported a detailed antitumor mechanism of these compounds using a murine model of colon cancer. The results demonstrated that 4-KLH could elicit IgG antibodies which could lyse STn-expressing cancer cells, stimulate antigen-specific T lymphocytes and activate both cellular and humoral immune responses, thereby breaking immune tolerance and generating the anti-tumor immune responses.

Analysis of the cellular and humoral immunity revealed that immunization with 2-KLH and adjuvant was less efficacious in antigen specific cellular immune response, but provoked a stronger STn-specific antibody response compared with 1-KLH. Humoral immune response is important for anticancer immunotherapy [37] and antibodies against TACAs can eliminate circulating tumor cells and micro-metastases [3]. However, only a strong humoral immunity without sufficiently strong cellular immunity may not be sufficient to cause antitumor effect in this study. The mice immunized with 3-KLH induced a weak cellular immunity and could not provoke a strong STn-specific immune response in comparison with 1-KLH. Immunization with 4-KLH produced a significantly stronger cellular and humoral immunity than that of 1-KLH, including a significant increase in the number of IFN-γ-releasing splenocytes, a greater cytotoxicity, and higher anti-STn IgG antibody titers. Next, we found that 4-KLH produced a mixed Th1/Th2 response, and produced Th1 response in an earlier stage than 1-KLH. Our results are consistent with previous reports, revealing that a potent anti-cancer vaccine should be able to initiate various actions of the immune system at once [38].

Incorporating an adjuvant into a vaccine can determine the immunophenotype and achieves qualitative alteration of the immune response [39]. To study the influence of carbohydrate hapten to the glycoprotein vaccine, we evaluated the effect of the vaccine in the absence of adjuvant on the immune response of tumor-bearing mice. Vaccination with 2-KLH induced mainly Th2 immune response after the third immunization and provoked a mixed Th1/Th2 response after the fifth immunization. Failure to induce an earlier Th1 response may be a limit to the effective anti-tumor activity. Vaccination with 3-KLH induced a weak immune response in comparison with 1-KLH. Vaccination with 4-KLH in the absence of adjuvant led to a significant reduction in tumor burden of lungs compared with 1-KLH and elicited a quantitatively higher and more rapid both Th1 and Th2 responses than 1-KLH. In the absence of adjuvant, 4-KLH can also cause a strong antitumor immune response, illustrating the structure modification can essentially improve immunogenicity and promote anti-tumor immunotherapy effect.

According to previous reports, CDC is a potent mechanism of cell killing [40], and CDC induction is associated with prolonged survival time in phase I trial [41]. ADCC can directly induce a variable degree of immediate tumor destruction, leading to antigen presentation and the induction of tumor-directed T-cell responses [42]. Therefore, selective CDC and ADCC activities against tumor cell lines are direct measurements of functional systemic anticancer immunity. For CDC and ADCC assays, we found that the antisera obtained by immunization with 4-KLH, whether in the presence of adjuvant or not, were able to increase the lysis of STn positive tumor cells compared with the 1-KLH group. It may be associated with that 4-KLH produced higher anti-STn IgG antibodies and induced more abundant IgG subtypes than 1-KLH.

The above results indicated that the fluorine modification strategy could improve the immune responses with or without adjuvant. Thus, 4-KLH may be a better anti-tumor immunotherapy candidate than STn-KLH, which was tested in clinic (Theratope) and did not induce significant therapeutic efficacy in patients [8]. The reasons for the enhanced immune response of fluoro-substituted STn are presumed as follows. T lymphocytes and B lymphocytes undergo a negative selection process against self-antigens, resulting in immune tolerance to self-antigens [43]. Incorporation of fluorine atoms into STn could make the derivatives to be non-self to the immune system and escape immune tolerance induction, so it might induce a strong immune response. Meanwhile the antibodies these derivatives induced could still cross-react with the native STn due to the similarity in atom radius and lipophicity of fluorine to hydrogen. Furthermore, it has been reported that the carbohydrate epitope binds the MHC molecule in conjunction with a carrier protein-derived peptide and stimulates the carbohydrate specific T cells [44, 45]. Chemical modification of tumor-associated antigens could improve the stability of MHC-peptide-TCR complex and enhance expansion of T cells specific for the natural tumor epitope *in vivo* [29]. Thus, fluorination of STn may improve the immune response by increasing the stability of MHC-peptide-TCR complex. As the electron-withdrawing nature of fluorine could influence the reactivity of the adjacent glycosidic bond [25], the glycosidic bond between the modified STn and peptides may be more stable against enzymatic degradation.

Overall, our study provides convincing results that the glycoconjugate 4-KLH established strong antitumor effects in murine colon cancer model and the antitumor effect of 4-KLH might be associated with the strong cellular and humoral immunity, which ultimately enabled the long lasting protection against tumor challenge. We are the first to demonstrate that the fluoro-modified glycoconjugate vaccine in which both the *N*-acetyl groups of STn are substituted is effective in a murine cancer model. The consequences from our research are very encouraging as they lay a foundation for improving the efficacy of STn-KLH and prove the feasibility of the fluorine modification strategy to the development of carbohydrate-based anticancer vaccines, thus holding the potential for effective cancer immunotherapy.

## MATERIALS AND METHODS

### Cell lines and culture

Mouse colon carcinomas cells CT-26 (STn positive) were obtained from Chinese Academy of Sciences (Beijing, China) and B16 cells (STn negative) were obtained from Chinese Academy of Medical Sciences (Beijing, China). Human colon carcinomas cells LS-B (STn negative) and LS-C (STn positive) were kindly provided by Dr. Steven H. Itzkowitz [46]. The STn antigen expression on the cells surface of the above mentioned cells was detected using an anti-STn polyclonal antibody by FACS (Supplementary Figure 3). The LS-B and LS-C cells were cultured in DMEM medium containing 1% (v/v) streptomycin-penicillin and 10% (v/v) fetal bovine serum (Hyclone), whereas the CT-26 and B16 cells were cultured in RPMI-1640 medium. All the cells used in this study were within 15 passages after receipt or resuscitation.

### Glycoconjugates

STn (Figure 1A, compound 1) and the modified derivatives 2 (with the 2-*N*-acetyl group of Tn moiety substituted by 2-*N*-fluoroacetyl group), 3 (with the 2-*N*-a cetyl group of Tn moiety substituted by 2-*N*-difluoroacetyl group), 4 (with the two *N*-acetyl groups of STn substituted by two *N*-fluoroacetyl groups) were synthesized by our laboratory. Carbohydrate-KLH and carbohydrate-BSA (bovine serum albumin) conjugates were synthesized by reductive amination reaction [17]. The epitope ratios of the glycoconjugates were determined by estimating protein content by BCA (bicinchonininc acid) assay [47] and sialic acid content with the resorcinol method [48]. The carbohydrate loading of glycoconjugates was shown in Supplementary Table 1.

### Immunization and immunotherapy of mice

The female BALB/c mice (age 6–8 weeks, No. SCXKjing2012-0001, SPF/VAF, purchased from Peking University Health Science Center) were randomly divided into five groups. One day before the immunization schedule, mice were given intravenous injection of cyclophosphamide (Sigma) at a dose of 100 mg/kg per mouse as the low-dose cyclophosphamide could reduce T-regulatory cells and enhance the immune response [49]. Mice were subcutaneously (s.c.) injected with a mixture of the adjuvant (First with Freund's complete adjuvant, and then Freund's incomplete adjuvant, Sigma) and the glycoconjugate vaccine modified-STn-KLH or STn-KLH in PBS (containing 2 μg of carbohydrate) on day 1. In the negative control group, mice were immunized with a mixture of adjuvants and KLH. Each animal received a total of five immunizations at biweekly intervals. At one week after the fourth immunization, all mice were challenged with 100 μL of 2 × 10^6^ cells/mL CT-26 tumor cell suspended in PBS by i.v. injection into the tail. The mice were bled by tail vein prior to the initial immunization and on day 13 after the third immunization. The blood was clotted to obtain sera, stored at –80^°^C. On day 70, the mice were bled and euthanized for a series of analysis described below. Animals used in this paper were well cared for and approved by Peking University Health Science Center.

### IFN-γ ELISPOT (enzyme-linked immunosorbent spot) assay

The IFN-γ-producing splenocytes were detected by ELISPOT mouse IFN-γ kit (Mabtech) referred to our previously established procedures [19] with minor modification. The 96-well PVDF-backed microplates were coated with a monoclonal antibody specific for mouse IFN-γ overnight at 4°C. The plates were washed with PBS and blocked with 10% FBS (in RPMI-1640 medium). Two weeks after final immunization, the mice were euthanized and their spleens were collected for the preparation of splenocytes after lysis of the red blood cells with 0.84% ammonium chloride. These splenocytes (1 × 10^6^ cells/well) were cultured with the corresponding glycoconjugates (0.2 μg of carbohydrate/well) and interleukin-2 (IL-2, 100 U/mL) at 37°C for 19 h. Then, the plates were washed with PBS and incubated with biotinylated monoclonal antibody specific for mouse IFN-γ for 2 h at room temperature. After washing, streptavidin–alkaline phosphatase was added and incubated with the plates for 1 h at room temperature. Then the plate was washed and a substrate solution of 5-bromo-4-chloro-3 indolylphosphate *p*-toluidine salt (BCIP)/nitro blue tetrazolium chloride (NBT) was added for 1 h at room temperature. The reaction was terminated with deionized water. After dried by air, the number of spots was counted using Immunospot Analyzer.

### CTL assay

Splenocytes (1 × 10^6^ cells/well) were cultured with the corresponding glycoconjugate for 24 h, then used as effector cells for CTL assay. Target cells CT-26 were treated with 5 ng/mL of IFN-γ (Pepro Tech) one day prior to the assay to up-regulate MHC class I surface expression. Effectors and targets at 100:1 were co-incubated for 24 h. At last, the effector cell-mediated cytotoxicity to target cells was examined by lactate dehydrogenase (LDH) assay according to the manufacture's protocol (Promega). Briefly, each plate was centrifuged at 250 g for 4 min, then 50 μL of the cell-free supernatant was carefully transferred to the corresponding wells of another 96-well enzymatic assay plate containing 50 μL of LDH assay reagents in each well. The plates were incubated at room temperature protected from light for 30 min, then 50 μL of the stop solution (1 M acetic acid) was added to each well of the plate. The absorptions of these plates were read at 490 nm wavelength using a microplate reader. In the meantime, the spontaneous LDH release values were determined by incubating tumor cells alone or splenocytes alone, respectively. The maximum LDH release values were determined by incubating tumor cells in RPMI-1640 containing lysis solution.

The percentage of cell lysis was calculated according to the following formula:

$$\%\text{Cytotoxicity}=\frac{\text{Experimental}-\text{Effector Spontaneous}-\text{Target Spontaneous}}{\text{Target Maximum}-\text{Target Spontaneous}}\times 100\%$$

### ADCC assay and CDC assay

Target cells LS-C (25 μL, 2 × 10^5^ cells/mL) were seeded in U bottom 96-well plates and incubated with test sera (25 μL, diluted 1/10 in RPMI-1640) at 37°C for 2 h. After unbound antibodies were removed, peritoneal macrophages isolated from healthy mice were added as effectors with an effector/target cell ratio of 10:1 (or 20-fold diluted rabbit complement serum for CDC assay) and incubated at 37°C for another 18 h. Then the cell supernatants were isolated and used to detect cell lysis by LDH assay as described above.

### Serological assay

Sera were tested as described previously for anti-STn and anti-modified-STn antibodies by ELISA [17]. Briefly, one hundred microliters of glycoconjugate STn-BSA or modified-STn-BSA (including 0.02 μg of carbohydrate in 50 mM bicarbonate buffer) were added to the 96-well ELISA plate and incubated for 1 h at 37°C (or overnight at 4°C). After washing with PBST, the ELISA plate was blocked with 3% BSA (200 μL/well) at 37°C for 1 h. The original serum was serially diluted with 1% BSA. The diluted serum was added to the plate (100 μL/well) and incubated for 1 h at 37°C. Then the plate was washed and incubated with horseradish peroxidase-conjugated goat anti-mouse IgG, IgG1, IgG2a, IgG2b, IgG3 or IgM (100 μL, diluted 1/5000) for 1 h at 37°C. At last, the *o*-phenylenediamine (OPD) substrate was added in the plate in the dark for 15 min, terminated by 2 M H_2_SO_4_, and then read at 490 nm. The antibody titer was defined as the highest dilution showing an absorbance of 0.1, after subtracting background.

### Flow cytometry

Sera were tested by flow cytometry. The assay was performed according to our previously established procedures [17, 19]. Briefly, tumor cells (5 × 10^5^ cells/tube) were washed in PBS with 3% fetal bovine serum and incubated with test serum (25 μL, diluted 1/20) for 30 min on ice. For the competitive inhibition assay, the test serum was mixed with STn (1.3 mg/mL) before it was added to the tumor cells. Then, goat anti-mouse IgG antibody labeled with FITC (25 μL, diluted 1/25) was added. Percentage positive cells and mean fluorescence intensity (MFI) of stained cells were analyzed using a FACScan (Becton Dickinson).

### Statistical analysis

Unpaired *t*-tests were used to analyze the antibody titers and lungs weight data. Log-rank test (Mantel-Cox) analyses were applied to evaluate the data from animal survival experiments. One-way ANOVA analyses were performed to evaluate the data of ADCC assay, CDC assay, ELISPOT assay and CTL assay experiments. *P* < 0.05 is considered as statistically significant. We used SPSS 13.0 for statistical analysis and GraphPad Prism 5 for graphics.

## SUPPLEMENTARY MATERIALS FIGURES AND TABLE



## Abbreviations

ADCC: Antibody-dependent cell-mediated cytotoxicity
BCA: Bicinchonininc acid
BSA: Bovine serum albumin
CDC: Complement-dependent cytotoxicity
CTLs: Cytotoxic T lymphocytes
ELISA: Enzyme-linked immunosorbent assay
ELISPOT: Enzyme-linked immunosorbent spot
KLH: Keyhole limpet hemocyanin
LDH: Lactate dehydrogenase
MHC: Major histocompatibility complex
MOE: Metabolic oligosaccharide engineering
SD: Standard deviation
SEM: Standard error of measurement
STn: Sialyl Tn
TACAs: Tumor-associated carbohydrate antigens
TCR: T-cell receptor
TF: Thomsen–Friedenreich
Th: T helper
Tn: Thomsen-nouveau
