# Supplementary material for: A cancer vaccine based on fluorine-modified sialyl-Tn induces robust immune responses in a murine model
Source: Oncotarget. 2017 May 7;8(29):47330–43. doi: 10.18632/oncotarget.17646 (PMC5564568; doi:10.18632/oncotarget.17646)
Supplement: Supplementary file 1 [file oncotarget-08-47330-s001.pdf]

A cancer vaccine based on fluorine-modified sialyl-Tn induces robust immune responses in a murine model

Supplementary Materials

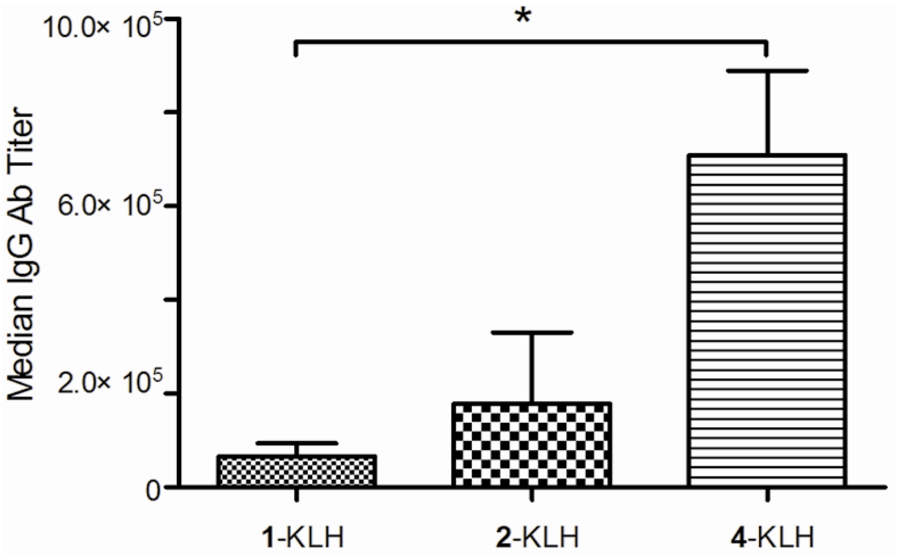

Supplementary Figure 1: ELISA anti-STn antibody titers after third immunization with antigen in the presence of adjuvant. ELISA plates were coated with STn-BSA and the antibody titer was defined as the highest dilution showing an absorbance of 0.1, after subtracting background. Results are expressed as the mean of individual ± SEM; $*p < 0.05$ using *T* test. The results are representative of three independent experiments.

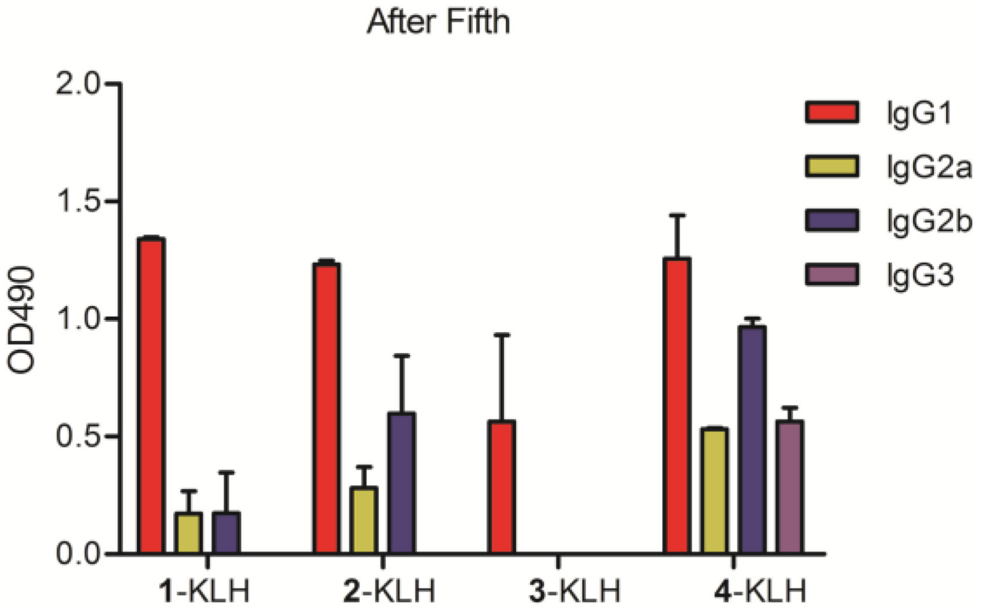

Supplementary Figure 2: IgG subtypes after fifth immunized with 1-KLH, 2-KLH, 3-KLH and 4-KLH in the presence of adjuvant by ELISA with a 1:1000 dilution of pooled sera. The pooled sera obtained from 14 days after the 5th vaccination. OD490 = optical density at 490 nm. Results are expressed as the mean ± SEM of at least two experiments.

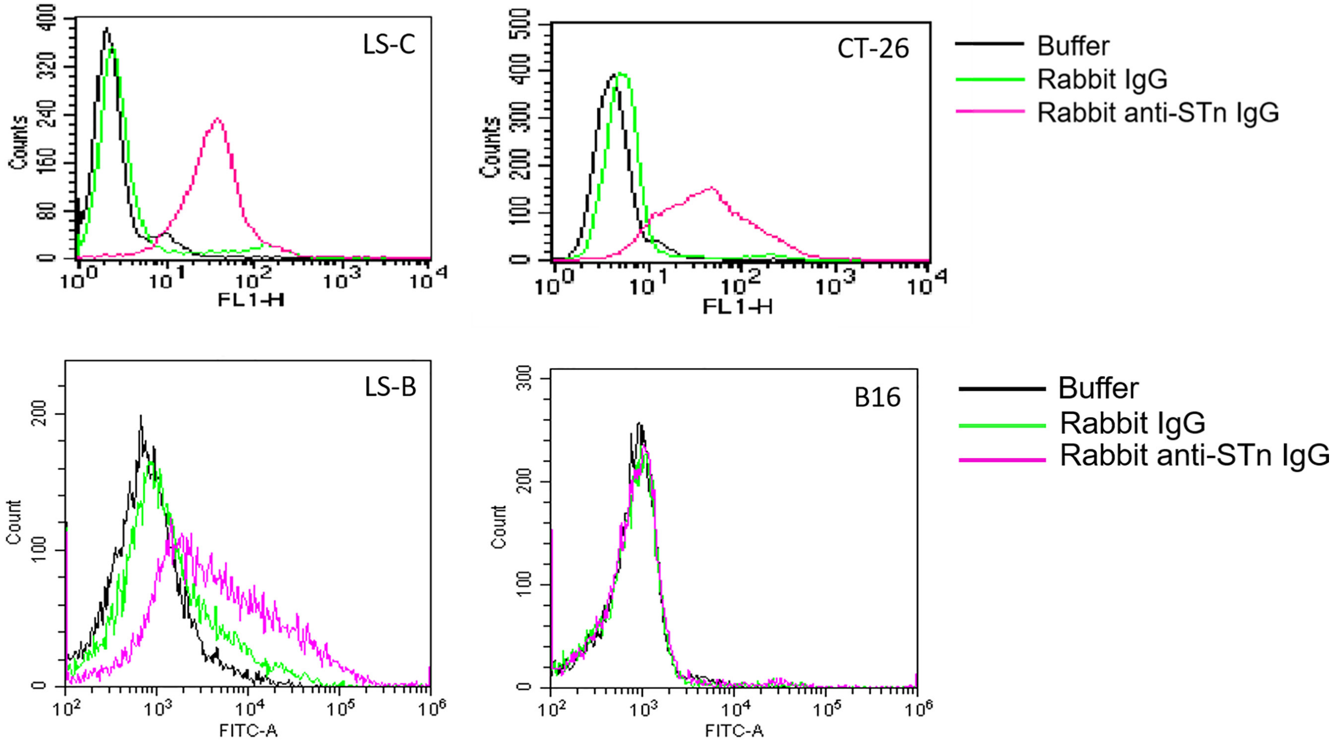

Supplementary Figure 3: Serological IgG analysis results on cancer cells by flow cytometry. The rabbit anti-STn IgG was used and tested against STn-positive cells (LS-C and LS-B human colon carcinomas cells; CT-26 mouse colon carcinomas cells; B16 mouse melanoma cells). Rabbit IgG and buffer were used as negative control. Rabbit anti-STn IgG polyclonal antibody was prepared by our laboratory. The results are representative of two independent experiments.

Supplementary Table 1: Carbohydrate loading of glycoconjugates

Sample	KLH conjugates loading (%)	BSA conjugates loading (%)
**1**	1.73	5.59
**2**	1.75	7.93
**3**	2.05	4.73
**4**	2.82	8.97
